# Bile Microbiota in Liver Transplantation: Proof of Concept Using Gene Amplification in a Heterogeneous Clinical Scenario

**DOI:** 10.3389/fsurg.2021.621525

**Published:** 2021-03-16

**Authors:** Francesco D'Amico, Alessandra Bertacco, Michele Finotti, Chiara Di Renzo, Manuel I. Rodriguez-Davalos, Gabriel E. Gondolesi, Umberto Cillo, David Mulligan, John Geibel

**Affiliations:** ^1^Hepatobiliary Surgery and Liver Transplantation, Department of Surgery, Oncology and Gastroenterology, Padua University, Padua, Italy; ^2^Transplantation and Immunology Section, Department of Surgery, Yale University, New Haven, CT, United States; ^3^Transplantation Unit, Intermountain Medical Center, University of Utah, Salt Lake City, UT, United States; ^4^General Surgery and Multivisceral Transplantation Unit, Department of Surgery, Buenos Aires University, Buenos Aires, Argentina

**Keywords:** microbiome, biliary infections, 16S ribosomal RNA gene amplification, rDNA sequence analysis, gallbladder

## Abstract

**Objective:** Historically, bile in the biliary tract has been considered sterile. Most of the series are based on patients with biliary tract diseases or the bile has been obtained with procedures susceptible to contamination.

**Methods:** We evaluated the bile in a heterogeneous cohort of liver donors and recipient patients, with samples obtained in a sterile way, directly from the gallbladder and the common bile duct.

**Results:** We assessed the bile microbiota in six liver donors and in six liver recipients after whole or split liver procedures in adult or pediatric recipients. Bile samples were studied using PCR sequencing of the 16S ribosomal RNA gene amplification (rDNA).

**Conclusions:** We demonstrated that the bile is sterile, thereby ruling this out as a source of contamination following transplant.

## Introduction

The gallbladder has been considered a very hostile territory for bacteria. Bile composition (bile acids, cholesterol, phospholipids, and the pigment biliverdin) functions as a biological detergent that emulsifies lipids. This property confers potent antimicrobial activity, primarily through the dissolution of bacterial membranes. For this reason, it has been assumed that the biliary tract is sterile in healthy individuals. Recent studies reported the presence of bacteria in other regions until now considered sterile including but not limited to (a) the female reproductive tract and (b) the urinary and respiratory tract (1, 2). Bacteria have predominantly been cultured from bile under these types of disease conditions. Bile compositions have been evaluated in patients with acute cholangitis and cholecystitis and recently in patients with distal cholangiocarcinoma (3). The bacteria most commonly include bacteria found in the intestinal tract, such as *Escherichia coli, Klebsiella, Enterobacter, Pseudomonas*, and *Citrobacter* spp.; their detection has been associated with progression to severe cholangitis and higher mortality rates (4). In particular, multiple enteric bacteria have developed defensive mechanisms conferring bile acid resistance (5). These mechanisms have been particularly studied in pathogens that are known to asymptomatically colonize the gallbladder, such as *Salmonella* spp. and *Listeria monocytogenes* (5–9).

An old previous study described—using electron microscopy, culturing, and detection of bacterial DNA—the identification and presence of bacteria in gallstones in non-inflammatory conditions (10). Recently, it became evident that the biliary system seems to have a complex microbiota, also in non-pathogenic situations (11). Wu et al. (12) evaluated the bile from 29 patients affected by gallstone diseases and the feces from 38 healthy patients. Surprisingly, the bacterial diversity was higher in the biliary system than in the intestine. There was a large similarity between the intestinal and biliary microbiome, although some significant differences were present: the biliary tract contains lower levels of Bacteroidetes (one of the two major gut phyla together with Firmicutes) but higher levels of Proteobacteria, TM7, Tenericutes, Actinobacteria, Thermi, and Cyanobacteria, while no differences are detectable for Firmicutes. Similar strains of bacteria have been found in a study investigating the bile of 39 patients with primary sclerosing cholangitis (PSC) (13). However, the majority of the bile samples of the previous study were collected via endoscopic retrograde cholangiopancreatography (ERCP), making intestinal contamination both possible and likely.

Due to the fact that—similar to bacteria in feces—the majority of the biliary microbiota is presumably uncultivable, the bacterial density in the biliary system might be comparable with the microbiota in the proximal parts of the small intestine, and the presence of bacteria in bile and gallstones may explain why bile leakage or gallstones lost in the peritoneal cavity during cholecystectomy often cause severe infectious complications. It is important to keep in mind that these earlier studies described bile, mucus, or tissue obtained from gallbladders.

Jimenez et al. (14) showed that the gallbladder ecosystem of white pigs is mainly populated by members of the phyla Proteobacteria, Firmicutes, and Bacteroidetes.

However, the global bacterial diversity of bile and gallbladder from healthy hosts has not been assessed yet. Recently, liver disorders such as non-alcoholic steatohepatitis (NASH) or PSC have been correlated with alterations in gut microbiome; it is unknown which kind of microbiome is involved in this process. Again, a study in 1990 by Ikeda et al. obtained samples only from the gallbladder in multiple-organ donors, selected by cooperation of donor surgeon, and concluded that flushing it during the procurement does not contaminate other organs procured (15). The aim of this study was to assess the presence of microbiome in the biliary tree in non-pathological conditions.

## Materials and Methods

### Patient Enrollment

From December 2015 to March 2016, six multiorgan donors in cerebral death and six liver transplant (LT) recipients were enrolled in the study. Donors and recipients fulfilled the international indication and contraindication criteria for multiorgan donation and LT. Our intent, respecting the previous criteria, was to create as much as possible a heterogeneous population in donor and recipient patients, in terms of clinical conditions and scenario. The study was exempt from approval from an ethic's board; the study was approved by the institutional review board.

Selected donors were candidates for organ procurement due to an irreversible cerebral injury. Donors with a history of gallstones or previous ERCP were excluded from the study, in order to avoid procedures or conditions associated with possible biliary contamination or bacterial translocation from gut to the biliary tree.

Liver recipients were a heterogeneous group of patients with end-stage liver disease. Recipient populations were represented from pediatric to adult cases, with different severity of liver disease and disparate comorbidities. All conditions were noted prior to tissue harvest.

We collected the following data: for donors, we evaluated age, gender, body mass index (BMI), cause of death, antibiotics therapy, intensive care unit (ICU) stay before the harvesting, cultural positivity, and type of graft; for recipients, we considered age, gender, model for end-stage liver disease (MELD), etiology of cirrhosis, cold and warm ischemia time, antibiotics therapy prior and after LT, cultural positivity, and hospital stay.

All donors and recipients underwent a standardized microbiological assessment, which included bronchoalveolar lavage (BAL), blood cultures, urine cultures, and nasal and rectal swabs.

Recipients were followed up until the hospital discharge in order to assess any complications, infections, cultural positivity, and outcome.

In the postoperative course, all the transplant recipients received a standardized nutritional support.

### Protocol of Bile Fluid Collection

Collections of the bile samples were done following a strict protocol to ensure aseptic conditions, avoiding any possible microbial contamination.

Bile was aspirated with a sterile 10-ml syringe. Five to ten milliliters of bile was sufficient for each sample.

Fluid collection was performed with sterile techniques described below:

- In donors, we collected bile with a fine needle from the common bile duct (CBD) at the beginning of the procurement after abdominal exploration. Consequently, we harvested the whole gallbladder after clamping cystic duct and artery with clips for further culture investigation. After that, we started with the standard procedure to harvest the abdominal organs. After aortic cannulation and cross-clamping, liver grafts were flushed with 6–7 L of cold preservation solutions (Celsior solution) to wash out intrahepatic blood and content. The liver was then procured and stored in sterile ice.
- In recipients, bile was collected at two different times, described as follows:
  ◦ With a fine needle from the recipients' CBD at the beginning of the transplant; this was after abdominal exploration and before starting the hepatectomy.
  ◦ From stump graft's CBD at the end of the warm ischemia time: after the portal and arterial liver reperfusion and before bile duct anastomosis.

### Bile Culture Techniques and 16S rRNA Sequencing

Aerobic, anaerobic, and fungal organisms were carefully evaluated with comprehensive cultures. All samples were cultured in chocolate agar (35°C + −2°C in aerobic atmosphere), blood agar (35°C + −2°C in anaerobic atmosphere), MacConkey II agar (35°C + −2°C in aerobic atmosphere), and Sabouraud glucose agar (35°C + −2°C in aerobic atmosphere) for 18–48 h. All potential isolates were observed by optical microscopy to determine their morphology and Gram staining. Additionally, they were tested for catalase, oxidase, and coagulase activities (Pastorex™ staph-plus, Bio-Rad).

To increase the strength of the study and to improve the microbial identification, bile samples were studied using PCR sequencing of the 16S ribosomal RNA gene amplification (rDNA).

### Study Endpoint

Bile microbiome was assessed in non-pathological biliary tree conditions.

## Results

### Biliary Microbiota

A total of 24 bile samples from 12 patients were obtained.

Twelve samples were collected during liver harvesting from brain death donors (six samples from the CBD and six samples from the gallbladder). Twelve samples were collected from the CBD of native and transplanted livers in six recipients, with a strict aseptic protocol.

Despite different donors' and recipients' conditions in terms of epidemiology features, comorbidities, and surgical procedures, all bile samples resulted negative: no microorganism growth was isolated in bile culture. The results were confirmed with the PCR sequencing of the 16S ribosomal RNA gene amplification (rDNA): no bacteria growth has been identified.

### Characteristics of Patients

Donors' and recipients' characteristics are reported in detail in Table 1.

**Table 1:** Donors' and recipient's characteristics.

**Features**	**Case 1**		**Case 2**		**Case 3**		**Case 4**		**Case 5**		**Case 6**	
	**Donor**	**Recipient**	**Donor**	**Recipient**	**Donor**	**Recipient**	**Donor**	**Recipient**	**Donor**	**Recipient**	**Donor**	**Recipient**
Age	76	65	87	70	40	70	66	2	39	41	30	65
Gender	M	M	M	M	M	M	M	F	M	M	M	F
Cause of death/liver disease	SAH	HCV–HBV cirrhosis HCC	SAH	HCV cirrhosis HCC	SAH	Acute NASH	SAH	ReLT in PNF	SAH	HCV cirrhosis	SAH	HCV cirrhosis
High WBC prior sample	No	No	Yes 14,000	No	Yes 24,000	No	No	No	No	No	No	No
Positive cultures	No	No	No	No	Yes	No	Yes	Yes	No	No	No	No
ICU stays (days)	5	4	4	4	12	1	32	10	3	2	3	6
Antibiotics	Proph.	Proph.	Proph.	Proph.	Proph.	Multi therapy	Meropenem	Multi therapy	Proph.	Oxazolidinone	Proph.	Proph.
Type of graft	Whole liver		Whole liver		Whole liver		Left liver hyper reduced *ex situ*		Whole liver		Right split liver *in situ*	
CIT (min)	505		545		510		360		318		350	
WIT portal/total (min)	30/60		30/60		25/50		25/58		30/60		30/60	
PO complications or infection	No		No		Sepsis and MOF on POD 1		*Enterococcus faecium* from abdominal drainage		No		Portal thrombosis in POD 1	

*SAH, subarachnoid hemorrhage; ICU, intensive care unit; CIT, cold ischemia time; WIT, warm ischemia time; PO, postoperative; Proph., prophylactic therapy; WBC, white blood cell; HCC, hepatocellular carcinoma; HCV, hepatitis C virus; HBV, hepatitis B virus; NASH, non-alcoholic steatohepatitis; PNF, primary non-function; POD, postoperative day; MOF, multiple organ failure*.

Donors 1 and 2 were suboptimal donors due to old age (76 and 87, respectively) and long ICU stay with no signs of infections. High white blood cell (WBC) count, hepatitis B virus (HBV) positivity, and long cold ischemia time (CIT) (16) in donor 2 were the additional pathological features. Their livers were allocated in low MELD recipients with cirrhosis complicated with hepatocellular carcinoma (HCC). Outcome was favorable with short length of stay (LOS) and absence of infections.

Donor 3 was a suboptimal donor due to a series of aspects: long ICU stay (12 days), high WBC (24,000 μl), high sodium level (163 mEq/L), need of hemodynamic support (dopamine 5 gamma in continuous infusion), fibrosis F1 at the liver biopsy, positivity for *Acinetobacter baumannii* at BAL examination, and antibiotic prophylaxis with cephalosporin for 12 days. The recipient was a sick patient with NASH cirrhosis that developed a rapid MELD increase (MELD 45 from 19). He was prioritized in the national waiting list in the last 2 weeks. He died on postoperative day (POD) 1 due to sepsis and multiorgan failure.

Donor 4 was a suboptimal patient due to long ICU hospitalization (32 days), high sodium level (145 mEq/L), and one blood culture positive for *Staphylococcus haemolyticus* and *Candida albicans*, under control with antibiotic therapy. The graft was harvested with the *N*-acetylcysteine protocol, as described by D'Amico et al. (17). Partial graft (left liver hyper reduced *ex situ*) was allocated in a pediatric recipient (case 4) with primary non-function (PNF) due to portal thrombosis, pediatric ESLD (PELD) 35, and one blood culture positive for *Mycoplasma*. The post-LT course was uneventful, except for an *Enterococcus faecium* positivity in the abdominal drainage treated conservatively with antibiotic therapy.

Donor 5 was a standard young patient with no comorbidities, and the LT was performed with no complications and with a low CIT (318 min) in a high MELD recipient (MELD 33). Case 6 was performed with a right split liver from a standard young donor with no comorbidities. The LT was performed in a hepatitis C virus (HCV) patient with portal thrombosis. The subsequent POD was complicated with portal thrombosis on day 1, resolved with surgical revision.

## Discussion

Clinical experience demonstrates that cholangitis is sustained by pathogens coming from the small bowel (ascendants cholangitis), peritoneum (perforation), and skin (fistula), with or without the presence of gallstones. Many studies reported the presence of positive bile culture under the previous pathological conditions. In particular, bile culture has been found positive with a rate ranging from 9 to 42% in elective resected gallbladders and from 35 to 65% in acute cholecystitis (18, 19).

The presence of microbiome in the biliary tree in non-pathological conditions is still controversial. Previous studies in the literature evaluated the bile composition in animals model or in patients with biliary tree diseases (in cholangitis, acute cholecystitis, and cholangiocarcinoma and presence of gallstone) using mostly ERCP to collect bile samples (3, 20, 21). The presence of a biliary induced pathological condition and the use of ERCP were the main limitations of these studies: indeed, Mahafzah et al. demonstrated that ERCP was an independent factor associated with positive bile culture (22). The data were recently confirmed by Sung et al. in a cohort of patients with acute cholecystitis (20). Probably, ERCP creates a direct connection between the duodenum and biliary tree, allowing ascending bile contamination.

Again, the study in 1990 on multiple organ donor did not consider the main bile duct as a possible sample area; neither did it follow a restricted protocol of exclusion for bile contamination or evidence of stones in the history of the patient (15).

The aim of our study was to investigate the bile composition in patients with no biliary alterations and no previous biliary procedure, represented by multiorgan donors, and to evaluate the bile behavior in liver recipients during LT.

For the first time, our study demonstrated that in non-pathological biliary tree conditions the composition of the bile is sterile. This is the first study where bile samples were obtain directly from the bile tree, not using an endoscopic tool (such ERCP), thus avoiding the likely gut contamination (23).

Donors and recipients with a history of gallstones or ERCP were excluded from the study, in order to avoid procedures or conditions associated with possible biliary contamination or bacterial translocation.

Furthermore, to improve the sensibility and specificity of our study, bile samples were examined using PCR sequencing of the 16S ribosomal RNA gene amplification (rDNA).

Bile sterility was confirmed in donor patients who, in order to donate, have to be healthy and not have any biliary complications and/or infections ongoing.

The results were confirmed also in liver recipients, a high-risk population in terms of comorbidities and sickness.

Therefore, we showed that even in the presence of features suggesting high risk of concomitant and possible infections, bile is sterile; indeed, bile culture, even in patients with high WBC, positive blood culture, or long hospital stay, was negative. Importantly, results remained consistent in the absence or presence of different antibiotic therapies. The heterogeneity of the considered population gives a strong value to the results of the study.

To note, we evaluated the bile composition during complex surgical procedures, such LT, LT with right, or left split liver. The results remained consistent among low-risk liver recipients and recipients with high risk of morbidity and mortality (patients in national urgency due to PNF or with high MELD scores).

We also showed that no bacterial translocation from the recipient to the liver graft takes place during LT procedure.

In conclusion, we demonstrated that in non-pathological biliary tree conditions and in patients with other medical conditions not related to biliary tree diseases, the bile is sterile, thereby ruling this out as a source of contamination following transplant.

## Data Availability Statement

The raw data supporting the conclusions of this article will be made available by the authors, without undue reservation.

## Ethics Statement

Ethical review and approval was not required for the study on human participants in accordance with the local legislation and institutional requirements. Written informed consent for participation was not required for this study in accordance with the national legislation and the institutional requirements.

## Author Contributions

FD'A, JG, and AB: conception and design of the article, analysis, and interpretation of data. FD'A, CD, and MF: acquisition of data. AB, FD'A, and MF: drafting the article. GG, DM, MR-D, and UC: revising the article critically. FD'A and JG: final approval of the version. All authors contributed to the article and approved the submitted version.

## Conflict of Interest

The authors declare that the research was conducted in the absence of any commercial or financial relationships that could be construed as a potential conflict of interest.

## Abbreviations

BMI: body mass index
CBD: common bile duct
ECD: expanded criteria donor
ESLD: end-stage liver disease
ERCP: endoscopic retrograde cholangiopancreatography
HCC: hepatocellular carcinoma
ICU: intensive care unit
LOS: length of stay
MELD: model for end-stage liver disease
NASH: non-alcoholic steatohepatitis
NAC: *N*-acetylcysteine
POD: postoperative day
PNF: primary non-function
PSC: primary sclerosing cholangitis
SCD: standard criteria donor.
